# Chinese TikTok (Douyin) challenges and body image concerns: a pilot study

**DOI:** 10.1186/s40337-023-00829-5

**Published:** 2023-07-03

**Authors:** Shuchen Hu, Jasmine Gan, Victoria Shi, Isabel Krug

**Affiliations:** 1 Teachers College, Columbia University, New York, NY USA; 2 Melbourne School of Psychological Sciences, University of Melbourne Psychology Clinic, The University of Melbourne, Redmond Barry, Level 7, North Melbourne, Melbourne, VIC 3051 Australia

**Keywords:** TikTok, Social media, Body image, Eating disorders, Body dissatisfaction, China, Asia

## Abstract

**Background:**

Social media content on Western platforms promoting thinness, or *thinspiration*, has been found to negatively affect body image perception of users. Less is known about non-Western social media use and its effects on body image concerns. Chinese TikTok, known as Douyin, is a popular short video platform with 600 million daily active users. Recent trends on Douyin encourage users to demonstrate thinness through participation in ‘body challenges’. This paper argues that such content is comparable to thinspiration, however, to date hardly any research has been undertaken on these challenges. Thus, this pilot study aimed to analyse the content of three viral challenges and investigate their impact on Douyin users.

**Methods:**

Thirty most viewed videos were collected for three challenges (N = 90): the Coin challenge, the A4 Waist challenge, and the Spider leg challenge. Videos were coded for variables relating to thin idealisation, including thin praise, sexualisation and objectification, and analysed through content analytic methods. Video comments (N ≈ 5500) were analysed through thematic analysis, and main themes were identified.

**Results:**

Preliminary findings showed that participants who objectified their bodies to a greater extent expressed more negative body image concerns. In addition, comments on the videos had themes of thin praise, self-comparison, and promotion of dieting behaviours. In particular, videos of the A4 Waist challenge were found to incite more negative self-comparison in viewers.

**Conclusion:**

Preliminary findings suggest all three challenges promote the thin ideal and encourage body image concerns. Further research about the broader impact of body challenges is needed.

## Plain English summary

Social media plays an important role in shaping public perceptions of body ideals. Short-video platforms, such as TikTok, have further facilitated the dissemination and consumption of media content and popularised challenge videos that involve wide user participation. The aim of our study was to analyse the content of three viral Chinese TikTok (Douyin) body challenges (e.g., A4 Waist challenge—participants aim to fit their waist behind an A4 sheet of paper) and investigate their impact on Douyin users. We extracted 90 short videos featuring the three body challenges from Douyin using Python and up to 100 comments from each video for qualitative analysis. Our findings indicated that the overt thin-ideal messaging, objectification and sexualisation of bodies in these body challenges may contribute to upward appearance comparison and greater body dissatisfaction. The present study provided initial support for the detrimental effects of Douyin body challenges on body image for both viewers and content creators. Through this pilot study, we hope to inform further research on the relationship between appearance-oriented content on short-video platforms and body image concerns.

## Introduction

The use of Western social media sites such as Facebook, Instagram, and Twitter have been linked to body dissatisfaction and disordered eating in users globally [1–4]. Greater duration of time spent on social media sites has been found to be associated with heightened body image disturbance and eating concerns [5, 6]. In particular, young women who spend more time on social media may be more concerned about their body image because of negative self-comparison with peers [4, 7]. Additionally, exposure to thin-ideal images in traditional media has been shown to increase state self-objectification, weight-related appearance anxiety, negative mood, and body dissatisfaction in viewers [8]. However, most of the literature on the impact of social media to date has focused on Western countries, with little research available on the impact of social media in other parts of the world. Broadly, this study aims to investigate the impacts of Douyin, a Chinese short-video platform, on body image satisfaction and potential body image disturbance of its users.

Compared to traditional mass media, social media has been found to have a pronounced impact on body dissatisfaction as it encourages upwards appearance comparison (comparing oneself to a body perceived to be more attractive than one’s own) with both peers and celebrities compared to the previous mass media that mainly focused on celebrities [4, 9]. The predominance of visual media on video- and image-sharing platforms such as TikTok or Instagram combined with 'thin ideal' stereotypes by users who engage in interactive comment exchanges for the different images and videos, presents a potential explanation for the strong association between social media use and eating concerns [5].

Importantly, following the outbreak of COVID-19, researchers have theorised that an increased use of social media during the pandemic and concerns about health and fitness during COVID-19-related isolation may have increased the risk of developing eating disorders in vulnerable individuals [10, 11]. In particular, symptom escalation correlated with the pandemic was most commonly reported in individuals with confirmed eating disorder cases, young women, athletes and parents or carers [12].

Given the prevalence of social media use globally, with an estimated 4.26 billion users in 2021 [13], it is important to examine the effect of social media on body image in other countries. With 926.8 million users alone, China is the largest national social media market in the world, despite government censorship of Western social media sites such as Facebook and Instagram [14]. Despite this high number of social media users in China, limited attention has been given to the impact of local social media in Asian countries [15]. This points to a gap in the literature on the impact of social media on body image in China, which is especially concerning given that rates of clinical eating disorders among female undergraduate students in China have been found to be on par with equivalent populations in the US [16]. Furthermore, while regional comparisons show that the prevalence of eating disorders have historically been lower in non-Western countries, they are gradually catching up [17–19].

### Media and body dissatisfaction in China

Studies focusing on a Chinese population have found that traditional mass media reflects societal expectations to be thin, which creates perceived appearance pressure that is linked to body dissatisfaction and other risk factors (such as negative affect) for disordered eating among young women [20–23]. Specifically, a recent 12-month longitudinal study found that Chinese women perceived more societal pressure and stronger preferences to be thin from Asian media rather than Western media [23], echoing findings that Chinese adolescent girls with greater exposure to Asian mass media (relative to exposure to US media) were more likely to judge themselves as larger-bodied [24]. However, the impact of media may be affected by the cultural context, as well as factors such as socioeconomic status, modernisation, and urbanisation, all of which may influence body ideals in non-Western cultures [15, 25, 26].

### Douyin and the prevalence of ‘body challenges’

In the past decade, the rising trend of online videos encouraging viewers to undertake or participate in ‘body challenges’ showcasing users' thinness has drawn concern about their contribution to increased disordered eating behaviours [27]. These videos have been popularised on Douyin, known as TikTok outside of mainland China, a social media platform with 689 million monthly active Chinese users (as of January 2021). Popular challenges include the ‘A4 Waist’ challenge and the ‘100 Yuan’ challenge, which respectively encouraged users to show how their waists were narrow enough to fit behind a sheet of A4 paper, or that their wrists were narrow enough to be encompassed by a 100-yuan bank note [28, 29]. A recent challenge in March 2021 required participants, usually young women, to fit inside a child's t-shirt from Uniqlo, a Japanese retailer [30]. These challenges have been heavily popularised through celebrity participation on public broadcast television shows and social media sites [31].

Despite implications of the challenges on body dissatisfaction and disordered eating, it appears that only one study has investigated the impacts of Douyin challenges on users’ body dissatisfaction. Jackson et al. [31] examined the effects of the A4 Waist challenge in an experimental study. The study assessed gender differences in participants' familiarity with the A4 Waist challenge, body image experiences of participants who passed or failed the challenge, and participants' changes in state body dissatisfaction after viewing videos of thin peers passing the challenge [31]. The study found that participants who were exposed to imagery of peers successfully passing the challenge reported having increased body dissatisfaction at the end of the study than the control group. Outside of psychology, studies in the fields of sociology and communications have observed that mobile short-video platforms like Douyin are more engaging than older social media platforms due to their content diversity and recommendation algorithms [32], and that Douyin can have strong influences on users’ consumption patterns, lifestyles, and even social values [33].

However, while the few studies above investigated Douyin using experimental and survey methods, no study to date has undertaken a content or thematic analysis of the most popular Douyin challenges and assessed the videos, nor comments by other users in response to these videos. Therefore, there is a need for researchers to further investigate the content and themes of these challenges, as well as the potential impact of the most popular challenges on users of both sides of the screen: video creators and viewers. There is already an established body of literature using content analytic methods on *thinspiration* and *fitspiration* content on Western platforms including YouTube, Instagram, Tumblr, and Twitter [34–36]. Additionally, several studies used thematic analytic methods on this thinspiration and fitspiration [34, 37]. However, no study has used similar methods to study Douyin videos in the Chinese webosphere.

### The present study

The current study aimed to fill intersecting gaps in the literature by investigating the impact of social media, specifically short-video platforms like TikTok or Douyin, on body dissatisfaction in a non-Western, Chinese population, by examining the impact of these videos on both video creators and viewers. It aimed to act as a pilot study to assist the preparation of a larger and more comprehensive research on the relationship between these viral body image challenges, body image concerns and disordered eating behaviours.

The study utilised a two-part qualitative design which built on the methodology employed by Ratwatte and Mattacola [34]. In that study, researchers combined content analysis and thematic analysis to investigate the influence of fitspiration videos on YouTube and user perceptions of fitspiration. Content analysis is a quantitative analysis of codified qualitative data, while thematic analysis is an inductive generation of present themes in qualitative data. Given the sparse literature surrounding body dissatisfaction and Douyin, and challenge videos in particular, this mixed-methods design aimed to explore this relationship more comprehensively.

In the current study, analyses were performed on imagery from the videos interacting in three popular Douyin challenges: the A4 Waist challenge (participants aim to fit their waist behind an A4 sheet of paper), the Coin challenge (participants fit as many coins as possible on their collarbones), and the Spider leg challenge (participants adopt a spider-like pose). Thematic analysis was performed on (1) audio transcripts of voiceovers alongside video captions and text overlaid onto video footage, and (2) corresponding viewer comments under the videos [34]. Greater understanding of the impact of the Douyin ‘body challenge’ videos could shed light on the risk factors for disordered eating within social media and how this influence can be mitigated.

## Methods

### Selection of challenges and collection of data

Ninety videos of users interacting in the challenges were collected in June 2021 through challenge-specific hashtags on Douyin #A4腰 (A4 waist), #锁骨放硬币 (collarbone coin) and #蜘蛛腿 (spider leg), comprising of the top 30 most-viewed videos for each challenge. Information about the social demographics for both video subjects and commenters of the 90 videos were collected using Python from Douyin user profiles, including age, gender, location (province in China), and number of followers.

**Fig. 1:**
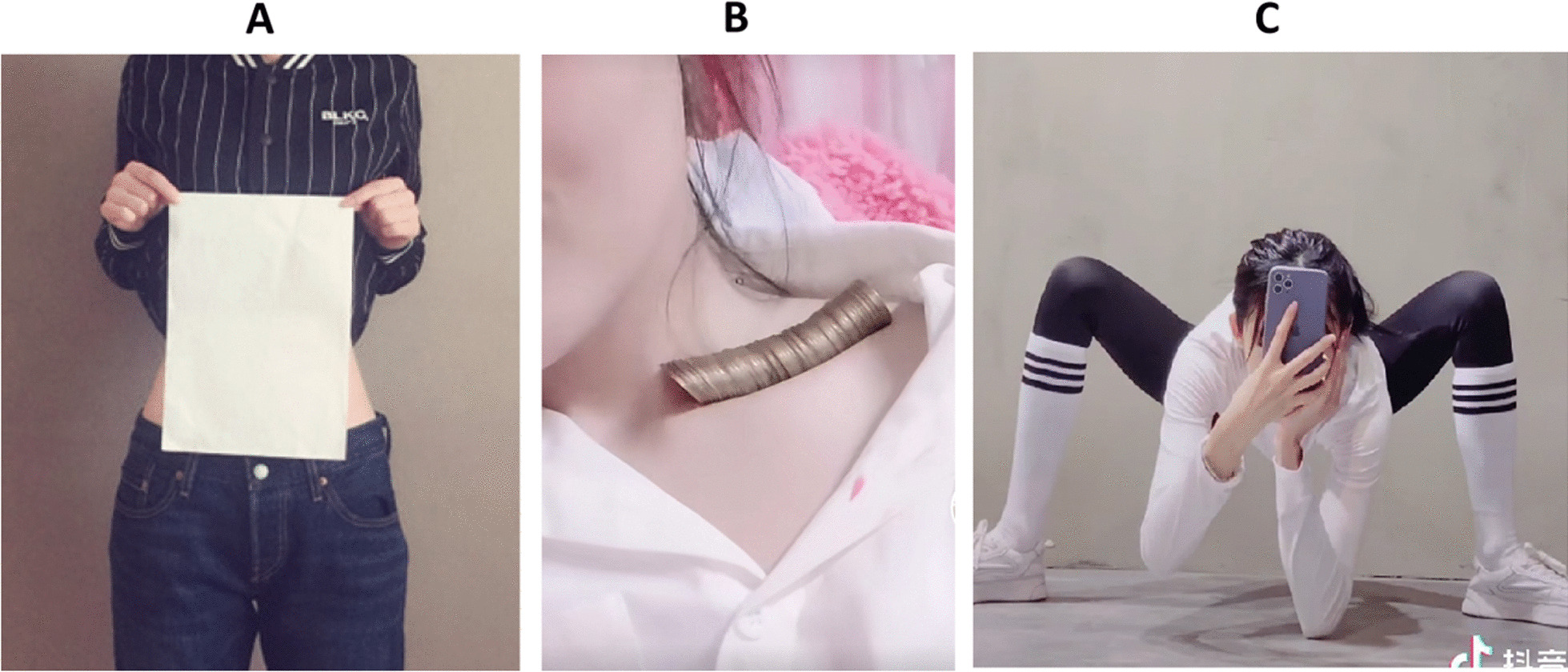
Examples of participants completing the challenges examined in the present study. *Note.* **A** depicts the A4 Waist challenge, in which challenge success is determined by a participant’s waist being completely obscured behind a standard-size A4 sheet of paper. **B** depicts the Coin challenge, a participant aims to fit as many coins as possible in their collarbone, challenge success is determined by a higher number of coins. **C** depicts a participant completing the Spider leg challenge, adopting the pose that displays their legs to resemble a spider. Here, challenge success is determined by the ability to touch one’s elbows to the ground

In the A4 Waist challenge, participants determine whether their waists can be completely hidden by a standard A4-size sheet. In the Coin challenge, participants aim to fit as many coins as possible in their collar bone. A higher number of coins is associated with a more prominent clavicle, a sign of thinness. Finally, in the Spider leg challenge, participants squat from standing until their elbows touch the ground, displaying their legs. A similar resemblance to a spider suggests thin legs. Figure 1 provides example pictures of these three challenges.

Demographic data was collated from extracted information of users’ online profiles, aside from gender. gender was coded by the authors based on the video subjects’ physical appearance.

### Part 1: Content analysis

Content analytic methods were utilised to analyse the extracted videos due to its ability to capture cultural nuances in the data through the coding of selected variables. Content analysis is defined as ‘a research method for the subjective interpretation of the content of text data through a systematic classification process of coding and identifying themes or patterns’ [38]. As videos and photographs have become more mainstream in the past decade, this method has been also used to analyse visual data [39]. In addition, it can encapsulate cultural contexts and linguistic nuances, providing a systematic way of reducing otherwise subjective data to focus on specific aspects of interest. A directed content analysis approach was chosen [38]; whereby the selection of initial codes was guided, using existing thinspiration and fitspiration social media studies [35, 36, 39, 40].

#### Codebook for content analysis

The coding scheme in this study was adapted from codebooks in the existing literature [35, 36, 39–41], and new variables were included to capture elements of Douyin and body challenge videos specifically. The main variable categories in the scheme assessed a range of descriptive attributes, appearance, body type and objectification, thin-ideal messages, and comparison, and mentions of food and exercise. Videos were collected by selecting the videos with the most views under each challenge’s unique hashtag. However, videos ‘tagged’ under the challenge did not necessarily show a creator participating in the challenge. Instead, some videos provided a critique or commentary of the challenge, while others provided physical exercise routines, ostensibly to aid challenge participants to succeed. Some video creators showed videos of several different clips of others participating in the challenge. Thus, descriptive variables were added to indicate whether the video creators themselves were actively participating in the challenge. Other new variables included the presence of appearance-modifying filters in the video, or the use of a recognisable music clip played over the video.

Variables assessing body type were operationalised as adiposity of video subjects, adapted from Tiggemann and Zaccardo [39], who examined visual content of Instagram fitspiration images. Objectification variables were operationalized as appearance of body parts in the videos, modified from Alberga et al. [35], whose study assessed messages relating to body shape from several social media platforms. Other studies have similarly utilised the proportion of body parts shown in the video as an indicator of body objectification [36, 41], as focusing on individual, decontextualised body parts dehumanises the subject as a person and the body is instead viewed as an object. Furthermore, sexually suggestive posing and clothing variables were adapted from Boepple et al. [40], who investigated body appearance-related themes in online fitspiration images. Variables relevant to ideals or guilt were based on verbal communications, both spoken by subject videos or written in captions, and non-verbals, such as posing and emphasising bony structures. Comparison variables were added and coded when subjects compared their appearances, either with themselves after engaging in exercise over a period of time, or alternatively with other people [36, 39, 42, 43]. Mentions of food and exercise were also coded, drawn from existing literature [36, 43].

#### Quantitative analysis of content

The variables were grouped into four categories based on the nature of each variable. The four categories were Video Attributes, Appearance of Video Subject, Objectification, and Thin Ideal. The team members (SH, JG, and VS) coded the videos individually, and discussed the results.

Variables were coded as present or absent. Two coders analysed each challenge, and the percentage of agreement and Cohen’s Kappa were calculated to assess the reliability of the coding. A third coder then resolved any discrepancies to create a final code. The frequencies of present variables were then used for quantitative analyses and comparison of video content, based on a method employed by Alberga et al. [35]. Differences in the frequencies of variables of interest across the challenges were not assessed using Chi-square analyses, or Fisher’s exact tests given that many of the counts were 0 for some of the challenges. Finally, descriptive statistics about the videos were collected, for example the percentage of videos with the challenge music, or the percentage of videos in which the subject themselves are speaking.

### Part 2: Thematic analysis

Thematic analysis methods were chosen to show the impact of challenge videos on viewers who commented on the videos, as well as the video creators themselves (who might be distinct from the video subject, that is, the individual(s) appearing in the videos, although in many cases they may be the same). Thematic analysis is defined as a method for identifying, analysing, and reporting patterns within data [44]. The advantage of this qualitative method is that no predetermined hypotheses are required, and it therefore reduces the influence of preconceptions in analysis [44]. Instead, themes and theories are inductively developed to uncover ‘context-phenomena relationships’ [45]. In this study, the context-phenomena relationship is the creation of appearance-centred challenge videos contributing to or reinforcing the phenomenon of body dissatisfaction in viewers. As this was a pilot study in a ‘natural experimental’ setting of real-world video viewers, these inductive methods assisted in the discovery of themes and patterns that have not yet been thoroughly established.

Phase one of the thematic analysis involves a general familiarisation of the dataset [44]. The three team members initially read through the comments of all three challenges, discussed and noted outstanding themes and trends.

For the second phase, initial codes were produced after a thorough review of the dataset [44]. The three members each analysed in-depth the comments for one challenge. In the initial stage, all potential themes were recorded. At various intervals of the coding process, the team shared and discussed noteworthy observations or patterns about the data, creating inductively ‘data-driven’ codes.

The third phase consisted of iterations refining and grouping codes into main themes [44]. Common codes were organised and structured as subthemes. An initial candidate map was created, and discussions were held to ensure all aspects of the data and codes were reflected in the identified themes.

The main overarching themes were developed in phase four and their relevancy and validity were confirmed by re-reading the datasets against these main themes. Relevancy ensures that data within a theme forms a ‘coherent pattern’ and no aspects of the data fall outside the main identified themes [44]. Secondly, the validity of the themes was assessed by considering whether the main themes reflected the dataset in its entirety. The themes were re-organised and re-structured until a satisfactory map was produced.

Finally, the essence of each theme was established and described (phase five) and sample comments were selected to demonstrate the themes (phase six).

#### Part 2a: Thematic analysis of audio transcript, captions and texts

Voiceovers in the videos were transcribed, and the audio transcripts were analysed alongside video captions or text overlaid onto video footage, using the process above. A separate thematic map was created. A random selection of 30% of the audio transcripts were cross-coded by a second coder for verification of themes. This allowed the observation of challenge impacts on video creators.

#### Part 2b: Thematic analysis of video comments

The top comments for each of the 90 selected videos were extracted using Python (N = 5508), capped at a maximum of 100 per video, and analysed for themes using the six-phase systematic process described by Braun and Clarke [44]. The comments were managed using NVivo 12 software [46]. Three team members (SH, JG, VS) who were fluent in Chinese and English coded the extracted comments.

A main purpose of this study was to inductively examine the prevalent themes within the viewers; thus, refined nuances were included in the map in the branched tiers to provide a more detailed picture of the viewers’ responses to the challenges. This also allowed contemplation of possible relationships between content (i.e., the videos) and the phenomena (the effect of viewing the videos on the users). The combination of content analysis and thematic analysis methods allowed us to observe the challenge impact from the perspectives of both the video subjects and the viewers.

## Results

### Sociodemographics of video subjects and commenters

Table 1 presents the demographics of the users (both video subjects and commenters) dataset and shows that more video subjects had female or feminine appearances. More commenters also identified as female, as indicated by their gender identity listed on their profiles with other personal information (e.g., age, location). While there was some missing data from the extracted information of their profiles on the gender identity of both the video subjects, the percentages of the female-appearing video subjects presumed to be the majority by observation. Female-appearing video subjects were in 93% of the Coin challenge videos, 93% of the Spider leg challenge videos and 87% of A4 Waist challenge videos. The dataset for commenters had more missing data as gender is not a requirement when signing up on Douyin, moreover, users have the option to keep their basic information private. Despite the limited information for commenters, more female-identifying individuals commented in the challenge videos compared to male-identifying individuals. All three challenges had more female-identifying commenters: the Spider leg challenge had 44% female-identifying commenters and 29% male-identifying commenters, the A4 Waist challenge had 39% female identifying commenters and 19% male-identifying commenters, and the Coin challenge had 31% female-identifying commenters and 21% male-identifying commenters. Particularly for the A4 Waist challenge, 39% of the commenters identified as female, nearly double the percentage of its male-identifying commenters (i.e., 19%).

**Table 1:** Collected demographic information about the videos and their corresponding comments

	A4		Coin		Spider leg	
	Video subjects (N = 30)	Commenters (N = 2571)	Video subjects (N = 30)	Commenters (N = 2710)	Video subjects (N = 30)	Commenters (N = 227)
Female Appearing or Identifying	26 (87%)	990 (39%)	28 (93%)	835 (31%)	28 (93%)	101 (44%)
Male appearing or identifying	4 (13%)	486 (19%)	2 (7%)	580 (21%)	2 (7%)	65 (29%)
Unknown	–	1095 (43%)	–	1295 (48%)	–	61 (27%)

Video subject gender data was collected from coders’ observations, while commenters’ gender data was collected from information extracted from commenters’ accounts

### Part 1: Content analyses

Table 2 summarises the variables used in the content analysis, further details about the variables are described below. The percentage of agreement for each variable was calculated based on the coding results for all three challenges. Overall, the agreement among the three coders was above high for most variables, with a few exceptions having less than 90% of agreement. These exceptions included challenge music (87% agreement), arms (87% agreement), body guilt (87% agreement), and thin pose (83% agreement) and filter (80% agreement) for the Coin challenge; and challenge music (67% agreement) for the A4 Waist challenge. The variables for the Coin challenge videos had less agreement overall among the three coders (SH, JG, and VS) compared to the other two challenges.

**Table 2:** Coded variables description and percentage agreement between three coders (SH, JG, VS)

Variable	Description	% Agreement		
		A4	Coin	Spider leg
*Video attributes*				
Participation in challenge	Video subject is participating in the challenge	97	100	100
Attitude towards challenge	Subject expresses earnest or serious attitude towards challenge	100	93	100
Gender appearance	Subject is masculine or feminine in appearance	100	100	100
Tagging another user	Caption tags another human user	100	100	97
Filter	Video uses discernible filter (including lighting filter, background filter, etc.)	97	80	97
Speaking	Subject speaks in the video rather than playing music or another sound	100	100	97
Other people	There is another person or people in the video other than the subject	100	97	93
Challenge music	Music used in video has been used with another video of the same challenge	67	87	100
Mention of celebrity	Captions or speech in video mentions a celebrity, or celebrity's images are used	97	97	100
Challenging others	Captions or speech in video encourage users to take up challenge	100	93	97
*Appearance of video subject*				
Thin	Subject of video is thin: slight frame with little to no visible fat stores	100	97	100
Average	Subject of video is average weight: medium frame with moderate level of visible fat	100	97	100
Overweight^a^	Subject of video is overweight: high level of excess fat	100	100	100
*Sexualisation*				
Suggestive pose	Subject adopts pose that emphasises sex characteristics	93	100	97
Revealing clothing	Clothing of subject reveals more than 70% of skin (eg. crop top and shorts, or less)	93	97	93
Lingerie	Clothing of subject resembles lingerie or underwear	97	90	100
Tight clothing	Clothing is tight-fitting in such a way that emphasises the subject's figure	90	97	100
*Objectification*				
Face visible	Face of subject is entirely visible and unobscured	100	97	100
Eyes	Eyes of subject are visible and unobscured	100	97	100
Full body or nearly full body visible	Full body or nearly the full body of subject is visible	97	97	100
Torso	Torso of subject is visible in video	100	90	100
Pelvis	Pelvis of subject is visible in video	100	100	100
Arms	Arms of subject are visible in video	100	87	100
Legs	Legs of subject are visible in video	97	100	100
*Thin ideal*				
Thin praise	Captions or speech in video include compliments for thinness	97	90	93
Thin pose	Posing or positioning camera to appear thinner or smaller	97	83	97
Bone emphasis	Prominent focus on bony features such as hip and collarbone protrusions	100	97	100
Before/after comparison	Video shows before and after weight loss comparison	100	100	100
Comparison with others	Subjects in video compares appearance with each other	97	93	100
Body guilt	Captions or speech in video expresses guilt for having gained weight or body type	10	87	100
*Other related variables*				
Dieting restraint messages	Captions or speech in video encourages dieting or reducing food intake	100	97	100
Mention of mental illness	Captions or speech in video mention eating disorder, self-harm, anxiety, suicide or depression	100	100	100
Mention of exercise	Captions or speech in video mention exercise	100	97	97
Mention of celebrity	Captions or speech in video mentions a celebrity, or celebrity's images are used	97	97	100
Challenging others	Captions or part in video encourage users to take up challenge	100	93	97

For each challenge, all the videos were coded by two coders, which gives the percentage agreement. The third coder then resolved any discrepancies to give a finalised code that is used throughout the study

^a^The original code for larger bodies was labelled as ‘overweight’ in this table and Table 3. However, ‘larger body’ is used throughout the paper to more accurately describe the observations made of appearance, as distinct from weight

Table 3 outlines video features and subject behaviours identified across all the three challenges and the frequency of their presence in a total of 30 videos for each challenge. The frequencies of each behaviour were recorded and presented as a percentage. The variables were grouped into six sections: Video Attributes, Appearance of Video Subject, Sexualisation and Objectification, Thin Idealisation and Other Related Variables.

**Table 3:** Frequency and percentage of variables and behaviours identified in each of the three challenges

Variable name	A4 (N)	Spider leg (N)	Coin (N)	Total (N)	A4 (%)	Spider leg (%)	Coin (%)	Total (%)
Participation in challenge	0	30	29	59	0.00	100.00	96.67	65.56
Positive attitude towards challenge	1	25	27	53	3.33	83.33	90.00	58.89
Gender (count of female appearing subjects)	26	28	28	82	86.67	93.33	93.33	91.11
Tagging another user	0	2	2	4	0.00	6.67	6.67	4.44
Filter	21	18	26	65	70.00	60.00	86.67	72.22
Speaking	5	6	5	16	16.67	20.00	16.67	17.78
Other people	7	3	1	11	23.33	10.00	3.33	12.22
Challenge music	12	29	12	53	40.00	96.67	40.00	58.89
Thin	21	28	22	71	70.00	93.33	73.33	78.89
Average	8	2	8	18	26.67	6.67	26.67	20.00
Overweight	0	0	0	0	0.00	0.00	0.00	0.00
Suggestive pose	18	2	1	21	60.00	6.67	3.33	23.33
Revealing clothing	5	3	3	11	16.67	10.00	10.00	12.22
Lingerie	1	0	2	3	3.33	0.00	6.67	3.33
Tight clothing	23	11	3	37	76.67	36.67	10.00	41.11
Face visible	17	13	16	46	56.67	43.33	53.33	51.11
Eyes	17	12	16	45	56.67	40.00	53.33	50.00
Full or nearly full body visible	8	30	0	38	26.67	100.00	0.00	42.22
Torso	29	30	4	63	96.67	100.00	13.33	70.00
Pelvis	26	30	0	56	86.67	100.00	0.00	62.22
Arms	24	30	5	59	80.00	100.00	16.67	65.56
Legs	8	30	0	38	26.67	100.00	0.00	42.22
Thin pose	24	0	13	37	80.00	0.00	43.33	41.11
Emphasis on bone	0	0	30	30	0.00	0.00	100.00	33.33
Before/after comparison	1	0	0	1	3.33	0.00	0.00	1.11
Thin praise	15	1	5	21	50.00	3.33	16.67	23.33
Comparison with others	4	0	0	4	13.33	0.00	0.00	4.44
Body guilt	6	0	2	8	20.00	0.00	6.67	8.89
Dieting restraint messages	0	0	0	0	0.00	0.00	0.00	0.00
Mention of mental illness	0	0	0	0	0.00	0.00	0.00	0.00
Mention of exercise	6	2	1	9	20.00	6.67	3.33	10.00
Mention of celebrity	2	1	2	5	6.67	3.33	6.67	5.56
Challenging others	0	2	8	10	0.00	6.67	26.67	11.11

Variables and Behaviours were divided into six sections based on their nature of characteristics. The sections in order describe the video content, body type, sexualisation, body image, thin idealisation, and others. Others include Dieting Restraint Messages, Mention of Mental Illness, Mention of Exercise, Mention of Celebrity, and Challenging Others. Among the six variables/behaviours, neither of the Dieting Restraint Messages or Mention of Mental Illness was found in any of the challenges

#### Video attributes

Video content variables included the presence of appearance-changing filters, use of audio clips that were associated with a particular challenge, and the use of video promoting strategies (such as encouraging viewers to participate in the challenges). All Spider leg challenge videos featured the subject participating in the challenge, 96.67% of the Coin challenge subjects participated, and none of the A4 Waist challenge videos featured the subject participating in the challenge. Similarly, subjects in most of the Coin and Spider leg challenge videos displayed a positive attitude towards the challenge (90.0% and 83.33% respectively), which was operationalised by an absence of criticism or satire in the video towards the challenge. On the other hand, only 3.33% of the A4 Waist videos displayed a positive attitude.

Most videos across all three challenges utilised a discernible filter that changed the subject’s appearance (86.67% of the Coin, 70.00% of the A4 Waist, and 60.00% of the Spider leg videos). Many videos also used an audio clip of music (‘challenge music’) that was recognisably linked to the particular challenge trend, with 96.67% of the Spider leg videos using the same music, and 40% of both the A4 Waist and the Coin videos also using such a music. Videos featured the subject speaking in 20% of the Spider leg, 16.67% of the A4 Waist, and 16.67% of the Coin challenge videos.

Lastly, only a small proportion of videos tagged another Douyin user in the caption, with 6.67% of the Spider leg and Coin challenge videos doing so, and none of the A4 Waist videos.

Percentages were calculated by dividing the total counts for each variable/behaviour by the total video count (N = 30). Total percentage was calculated by dividing the total counts for each variable/behaviour by the total video count for all videos collected (N = 90).

#### Appearance of video subjects

Video subjects had a thin body type in 93.33% of the Spider leg challenge videos, 73.33% of the Coin challenge, and 70% of the A4 Waist challenge videos. Average body type presentation was found in 26.67% of the A4 Waist and the Coin challenge, and 6.67% of the Spider leg challenge. Notably, video subjects of a larger body type were not coded for in any of the challenge videos.

#### Sexualisation and objectification

Sexualisation was operationalised through the variables of Suggestive Pose, Revealing Clothing, Tight Clothing across all three challenges. The A4 Waist challenge was associated with sexualising behaviours more compared to the other two challenges, as Suggestive Pose was found in 60% of the videos, Revealing Clothing was found in 16.67% of the videos, and Tight Clothing was found in 76.67% of the videos. In 3.33% of the A4 Waist videos, video subjects were wearing lingerie or undergarment-like clothing (‘Lingerie’), which was also found in 6.67% of the Coin challenge videos. None was found in the Spider leg challenge. Tight Clothing was found in 41.11% of all videos collected across the three challenges, which became the most represented sexualisation behaviour variable in this study.

Objectification was measured by the presentation of different body parts in most of the videos across all three challenges. Videos featured body parts of video subjects in most of the videos across all three challenges, but the predominant body part present differed according to each challenge due to the focus of different body parts in each of the three challenges. Among the seven body-part-related variables, including Face, Eyes, Full or Nearly Full Body Visible, Torso, Pelvis, Arms, and Legs, more than half of them (i.e., Face, Eyes, Torso, Pelvis, Arms) were found in more than 50% of the videos collected for all three challenges. Seventy percent of the videos had participants of the challenge with their torso visible, 65.56% had their arms visible, 62.22% had their pelvis visible, 51% had their face visible, and 50% had their eye visible. The visibility of legs was found in 42.22% of all videos and the visibility of full body or nearly full body was found in 42% of all videos.

#### Thin idealisation

A total of six behaviours were found to be associated with the idealisation of thinness: Thin Pose, Bone Emphasis, Before/After Comparison, Thin Praise, Comparison with Others, and Body Guilt. Subjects in a large proportion of the A4 Waist videos (80.0%) took up a thin pose, whereas only 43.33% of subjects in the Coin videos did so. In contrast, none of the Spider leg challenge videos featured a thin pose (by nature of the challenge, which required participants to take up another pose).

Thin Praise was found in 50% of the A4 Waist, 16.67% of the Coin, and 3.33% of the Spider leg challenge videos. The variable of Bone Emphasis was only observed in the Coin challenge, appearing in all of the Coin challenge videos and none of the other two challenge videos.

Only a small proportion of videos showed a before and after comparison of the video subject’s appearance (3.33% of the A4 Waist challenge only). Similarly, only 13.33% of the A4 Waist videos featured subjects comparing their own appearances to others.

#### Other variables

Other Variables include Dieting Restraint Messages, Mention of Mental Illness, Mention of Exercise, Mention of Celebrity, and Challenging Others. These variables were coded according to sentiments directly expressed by the video subjects (other sentiments expressed in the audio transcript, overlaying text and video captions were analysed using thematic analysis—see below). Among the six variables or behaviours, neither of the Dieting Restraint Messages or Mention of Mental Illness was found in any of the challenges.

Mentions of Exercise were found in 20% of the A4 Waist, 6.67% of the Coin, and 3.33% of the Spider leg challenge videos. Many videos with mention of exercise, particularly in the A4 Waist challenge, presented exercise coaching to help viewers pass the challenge.

Mention of celebrities was coded in 6.67% of the A4 Waist, 6.67% of the Coin, and 3.33% of the Spider leg challenge videos.

The Coin challenge featured the highest proportion of videos where users encouraged viewers to participate by filming themselves (26.67%), followed by the Spider leg (6.67%). In contrast, none of the A4 Waist videos challenged viewers to participate.

### Part 2a: Thematic map of audio transcript, captions and text

**Fig. 2:**
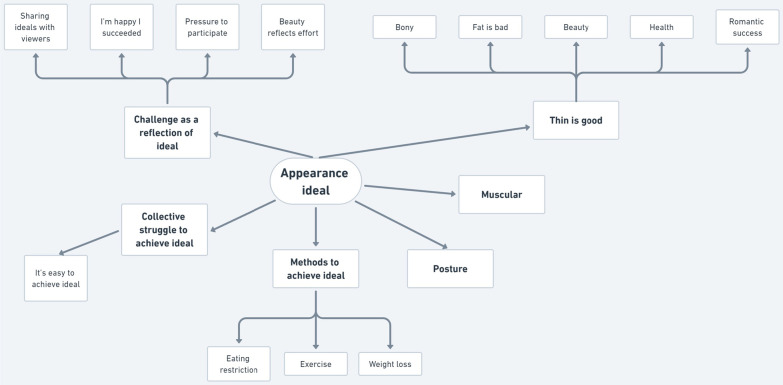
Map of themes in audio transcript, cations, and text. *Note.* After the extraction of the challenge videos, audio transcripts (voiceover audio) were analysed thematically with text overlaid on video footage and video captions. The major theme identified was Appearance Ideal, which could further branch into four themes with greater presentations and two themes that are relatively less presented. The four themes with greater presentations were Challenge as a Reflection of Ideal, Collective Struggle to Achieve Ideal, Methods to Achieve Ideal, and Thin is Good. Then each of the four themes were further branched into subcortical themes that reflect the audio transcript, captions, and text. The two themes with relatively less presentations in the transcripts were Posture and Muscle, and they did not have further branches or variations

Figure 2 presents the main themes found in the audio transcript, text overlaid over the video footage, and captions. The map extends outward from the centre theme Appearance Ideals, which further branched into four themes with greater presentations and two themes that are relatively less presented. The four themes with greater presentations were Challenge as a Reflection of Ideal, Collective Struggle to Achieve Ideal, Methods to Achieve Ideal, and Thin is Good. Each of the four themes further branched out into subthemes reflecting recurring patterns in the audio transcript, captions, and text. Two minor themes were Posture and Muscular, which did not have further branches or variations.

#### Challenge as a reflection of ideal

This subtheme reflected the idea that the challenges are a benchmark for an accepted ideal appearance. Video subjects reveal feeling pressure to participate (‘people wanted to see me do this challenge’), feeling happy at having succeeded (‘I didn’t think I would get this many coins!’). In several videos, video subjects implied that their viewers shared their appearance ideals, stating that ‘this is the body part that you want to see!’. Many video subjects also suggested that putting effort into improving the body shape will result in challenge success, and therefore achieving beauty ideals (‘Only these many reps a day will make you be able to succeed in the challenge!’).

听说你们都想我表演蜘蛛腿。(I’ve heard that you guys wanted me to perform the Spider Leg challenge) - SL25^1^

你们要的锁骨来了。看了千万别流口水。(The collarbones that you want are here. Please don’t drool.) - C1, C14, C17

哇哦!居然成功了 (Woah, I actually succeeded!) - C7

今天准备40个硬币挑战, 华晨宇的锁骨放硬币, 希望我能成功。(I’ve prepared 40 coins for the challenge today, the amount that Hua Chenyu^2^ put in his collarbone, I hope I succeed!) - C8

挑战一下看看能不能放更多的硬币。加油好好瘦! (Challenge yourself to see how many coins you get. Come on, get nice and thin! / I’m cheering for you to be thin!) - C15

#### Collective struggle to achieve ideal

This theme was presented in the sentiments that ‘we are all in this together’, and that ‘it’s easy/difficult to achieve the ideal [of a thin appearance]’.

这个动作啊, 大家就是看着觉得这个它太难了, 其实我觉得这个动作大家都可以做的到。(Everyone sees this action and thinks it’s too difficult, but I think it’s an action that everyone can actually do) - SL21

一个简单的动作燃烧全身脂肪 … 赶紧连起来! (A simple action to burn the fat off the whole body… don’t waste any time and go train!) - A19

这个夏天一起GET吧! (This summer, let’s GET it together!) - A30

#### Methods to achieve ideal

Several subjects presented informative videos on a range of eating restriction, exercise and weight loss advice either directly to succeed in the challenge or included the hashtag to leverage the challenge’s popularity to increase personal exposure. Interestingly, all mentions of eating restriction were not voiced by the video subject themselves but were present in the voiceovers.

每天睡前练一遍, 你的肚子腰线就会越来越漂亮了。 (Do this [exercise] once every day before bed, and your waistline will become more and more beautiful) - A4

坚持30天甩掉10斤小赘肉! (Keep it up for 30 days to lose 10 pounds of muffin top fat!) - A6

暑假大吃特吃, 然后肚子越来越大, 输家(sic)前买的衣服都穿不上了 (Ate a lot during the summer holidays, and your stomach got bigger and bigger, and the clothes you bought before summer don’t fit you anymore?) - A30

#### Thin is good

Many videos explicitly stated reasons for idealising thinness, including health, romantic success. Some commended certain features of thinness such as a bony appearance or denounced fat.

明天李现绯闻女友, 你来当。 ([After doing these exercises,] Tomorrow, you will be the rumoured girlfriend of Li Xian!^3^) - A1

你是不是像这样侧躺的时候呢, 发现腰线出来了, 但是肚子前面的肉呢, 却流到床上。 (Do you find that when you lie down, your waistline is more defined, but the rolls of your stomach flop onto the bed?) - A3

为啥你的腰粗想个大水缸? (Why does your stomach look like a big water pot?) - A18

男朋友更爱我了 (Your boyfriend will love you even more) - A30

### Part 2b: Thematic map of video comments

**Fig. 3:**
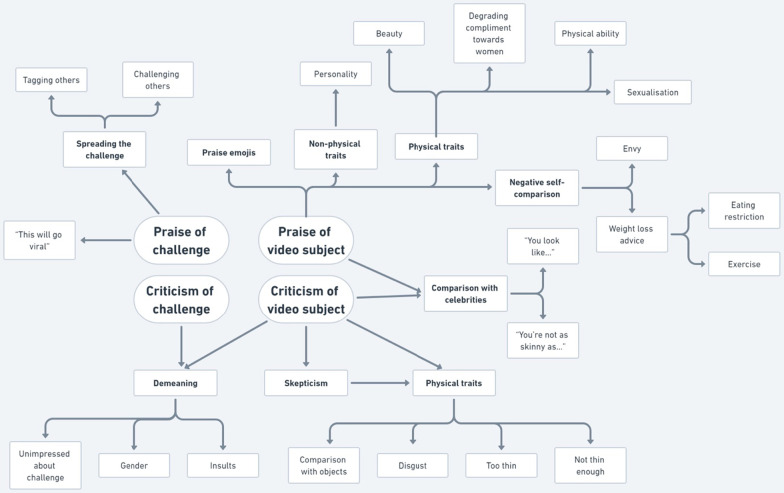
Map of comment themes. *Note.* The map is organised into a multi-layered structure from the most frequent themes to the least frequent themes from the centre going outward. Four overarching themes in the centre of the map were developed which could describe most comments (Praise of Challenge, Praise of Video Subject, Criticism of Challenge, and Criticism of Video Subject)

Figure 3 presents a thematic map connecting the themes of the comments in response to the videos of the investigated challenges on Douyin. The map demonstrates that most videos elicited comments falling into four main themes: Praise of Challenge, Praise of Video Subject, Criticism of Challenge and Criticism of Video Subject. Each of these themes contain secondary and tertiary themes providing nuances within the corresponding major theme, and praise of video subjects contains an additional level of themes as weight loss advice was further divided into eating restriction and exercise that reflected the existing literature for their associations to body image concerns and EDs [47, 48]. Among the four main themes, Praise of Video Subject and Criticism of Video Subject both had a secondary theme being Physical Traits, but their presentations were not entirely identical. The themes under Physical Traits for the Praise of Video Subjects include complimenting the subjects being beautiful, degrading compliments toward women (ie. “you have a great body to bear my children” or “you make a beautiful housewife”, physical ability (ie. “wow you can do this challenge/movement”), and sexualisation comments. The Physical Trait subtheme under Criticism of Video Subject included commenting that subjects are not thin enough or too thin, comparison with other people, and personal insults such as “you disgust me”. The themes Criticism of Video Subjects and Criticism of the Challenge shared one common theme, which is Demeaning Comments. Demeaning Comments could present themselves as personal insults, gender comparison, or commenter being unimpressed about the challenge that the video subject is doing.

#### Praise of challenge

Many comments expressed their approval of the challenge itself, seeing it as an instrument worthy of being spread and popularised. The most common type of comment was a user tagging another user.

@user 我想看你拍个这个 (@user I want to see you film this) - SL20.92

#### Praise of video subject

There were several notable subsets within praising video subjects. Approval of the subject’s physical traits was frequently observed. Another common method of praising video subjects was upward comparison, originally described by Festinger (1954). A small number of comments praised the subject’s non-physical attributes such as humour or personality.

##### Physical praise of subject

A subset of Praise comments directly expressed praise of the video subject’s physical features.

你的身材是我的目标 。I envy your body figure) - SL12.40

我(...马达啦...)愿称你为蜘蛛腿系列最美, 腿好长呀。 (I’m willing to say that your legs are the most beautiful of the spider leg series, your legs are very long) - SL5.1

个人喜欢王丽坤胯宽腰细才好看 (Personally, I like Wang Likun’s^4^ wide hips and thin waist, and one can only be beautiful with these features.) - A1.1

瘦下来的你, 更好看了【666】^5^ (You’ve gotten skinnier, you look much better! [“Cool1” emoji]) - SL29.85

##### Negative self-comparison

A subset of praise was indirect, taking the form of upwards comparison, which was often paired with a desire to restrict eating, or seek advice to lose weight.

看着她们的腰, 我默默放下了手里的汉堡 (Looking at their waists, I silently put down the hamburger that was in my hand) - A1.96

看完你所有的视频。我决定了吃完这顿再考虑减不减肥这问题 (After watching all of your videos, I decided to finish this meal and then consider whether or not I should go lose weight) - A12.82

我就想问问吃什么小腿可以比躯干长? (I want to ask, what do you eat to make your calves longer than your torso?) - SL6.34

小腿肚子都是肌肉怎么能瘦一点 (My calves and stomach is all muscle, how do I slim down a bit?) - SL29.29

#### Criticism of challenge

Comments conveying criticism of the challenge expressed concern for potential impacts of the challenge on society. However, another more prominent type of critical comments often belittled the value or difficulty of the challenge.

这是传播的什么文化? (What type of culture is this spreading?) - SL9.6

新时代裹小脚 (Modern day foot-binding) - A1.51

都在挑战这个动作, 可是这个动作又不难。 (Everyone is challenging themselves with this action, but the action isn’t even hard) - SL26.11

#### Criticism of video subject

Similar to praising the video subject, many comments criticising the video subject equally mentioned their physical traits. Many drew comparisons between the subject and an object, or other people.

抱歉, 姐姐, 我从来都没见过腿这么短的蜘蛛。 (Sorry sister, I’ve never seen a spider with such short legs) - SL4.30

## Discussion

The present study aimed to investigate the influence of three trending Douyin challenges (the A4 Waist challenge, the Coin challenge, and the Spider leg challenge) on body dissatisfaction, body image concerns, and disordered eating behaviours of social media users. The main findings of the content analysis suggest that, similar to thinspiration and fitspiration content on Western social media platforms [35, 39], Douyin body challenge videos further facilitate the internalisation of thin ideal and promote body guilt. Additionally, the findings from the thematic analysis of video voiceovers, captions and text suggest video creators fixate on appearance ideals. Viewers are highly likely to spread the challenge, engage in self-comparison, and comment on the physical appearance of subjects. Each of these findings will be discussed in more detail in the subsequent sections.

### Content analysis

In order to better present the uniqueness of Douyin, variables mentioning celebrities and challenging others were created as a new addition to the codebooks from previous literature, which included a range of descriptive attributes, appearance, body type and objectification, thin-ideal messages, and comparison, and mentions of food and exercise [35, 36, 39–41].

#### Body type and thin ideal

From the content analysis of the 90 Douyin videos, the predominant body type of video subjects was the thin body type across all three challenge videos. Video subjects who had a thin body type were present in 93% of the Spider leg challenge videos, 73% of the Coin challenge videos, and 70% of the A4 Waist challenge videos. More video subjects had thin body type in the Spider leg challenge videos as opposed to the other two challenges, which may be due to the nature of the Spider leg challenge that required participants to film their entire bodies (instead of, for instance, the participants’ torsos alone). The overall predominance of thin bodies in the videos aligns with findings from studies which performed content analysis on fitspiration and thinspiration content on Western social media sites and suggested that the Western female body ideal is one of thinness [34, 36, 43]. Our current findings therefore confirm the findings of other studies, which theorise that the contemporary Chinese female body ideal also incorporates thinness as a key attribute [49–51].

This suggests that the Douyin body challenge videos, as a form of social media designed to show off video subjects’ physical appearances, may enable appearance comparison in a similar manner to thinspiration and fitspiration content. Social comparison has been theorised as the major mechanism for media exposure to lead to body dissatisfaction and disordered eating through sociocultural exposures [52]. The most notable of these models is the Tripartite Influence Model [53] which proposes that the main influences for body image are peers, parents (or family more broadly) and the media. The Tripartite Influence Model has been further supported empirically in adolescent girls in the US [54], Asian women [55, 56] and across a number of demographic groups [56–58]. Thus, the Tripartite Influence Model can be tentatively extrapolated to Douyin viewers in China. It can be theorised that body challenge videos, and similar trends on social media more broadly, may negatively impact Douyin users’ body satisfaction by providing another avenue for users to engage in social comparison with their online ‘peers’.

Our findings also align with the only experimental findings of the impact of Douyin challenges on body dissatisfaction [31], which found that women with existing body dissatisfaction exposed to the A4 Waist challenge reported more significant increases in state body dissatisfaction compared to those exposed to control images (images of women that did not call attention to body size). Hence, our findings suggest that people with existing body dissatisfaction who then engage in body-focused Douyin challenges as either a subject or a viewer would be more likely to report greater body dissatisfaction.

#### Thin praise, thin pose, exercise and bone emphasis

Some proportion of the body challenge videos contained thin praise. Thin praise was found in 50% of the A4 Waist, 16.67% of the Coin and 3.33% of the Spider leg challenge videos. As above, this draws similarities with thinspiration and fitspiration images of women on Western social media sites. In particular, Tiggemann and Zaccardo [39] found that a majority (75%) of thinspiration and fitspiration images featuring women of a thin body type. On the other hand, of the thinspiration and fitspiration images showing men on Western sites, Tiggemann and Zaccardo [39] found that a majority (98%) were of men of an average body type, with 0% of a thin body type. However, in our current study, there were very few videos extracted featuring men (N = 8 in 90 videos). We were therefore not able to assess gender differences of the subjects in the Douyin challenge videos.

The discrepancy between frequency of thin praise in each of the challenges (50% in A4 Waist, 16.67% in Coin and 3.33% in Spider leg) may be explained by the nature of the challenges. As the Spider leg challenge required participants to perform a difficult pose (squatting like a spider), video subjects tended to speak more about the flexibility and physical ability to ‘succeed’ in the challenge. Thus, the focus was not solely on the physical appearance of the participants, as thinness itself was not sufficient to succeed. In comparison, the A4 Waist and Coin challenges only required participants to showcase their physical appearance, which may explain the higher frequency of thin praise in those challenge videos. However, despite the additional emphasis on participants’ physical ability in the Spider leg challenge, Spider leg videos still had the highest percentage of thin video subjects out of the three challenges (93.33%, as discussed above), even though ostensibly, thinness was not required to succeed. This may suggest that despite the superficial emphasis on physical ability, video subjects in the Spider leg challenge were primarily focused on their appearance and chose to participate to showcase their thinness.

The notion of displaying physical thinness through participating in the challenges is further bolstered by the findings relating to Thin Pose. A high proportion of video subjects in the A4 Waist challenge (80%) performed a thin pose. This finding is expected, as the A4 Waist challenge required participants to showcase their physical thinness. However, in the Coin challenge, almost half (43.33%) of video subjects performed a thin pose, although the challenge ostensibly did not require physical thinness to succeed. This may suggest that Coin challenge participants were conscious of their physical appearance while undertaking the challenge. (As expected, none of the Spider leg video subjects performed a thin pose, as the challenge required subjects to perform the ‘spider’ pose.)

Bone Emphasis was specific to the Coin challenge, given that the focus of this challenge was on one’s collarbones. Hence, this behaviour was found in all of the Coin challenge videos and did not appear in the videos of the other two challenges. This suggests that there is a strong similarity between the Coin challenge videos and *bonespiration*, a subset of thinspiration images glorifying bone protrusions found on Western social media sites such as Instagram, Tumblr and Twitter [35]. Hence, challenges that are also bone focused, such as the Coin challenge in the current study, may interact with body image issues in a similar manner as bonespiration images.

Mentions of exercise were coded in 20% of the A4 Waist, 6.67% of the Coin, and 3.33% of the Spider leg challenge videos. Research has consistently reported that exercise decreased body dissatisfaction and reduced body image concerns [59, 60]. However, mentions of exercise in the context of Douyin challenges may have a different trajectory. The exercises mentioned in all three challenges were meant to help viewers lose weight in the hope to complete the challenges by meeting the body standards. This is dangerous because it is indirectly promoting a thin ideal and normalising unrealistic body standards, which may have greater harm on a broader public as it is harder to be recognized, detected, and banned by Douyin regulations.

#### Sexualisation and objectification

Compared to the Coin and Spider leg challenge videos, a greater proportion of A4 Waist challenge videos were coded as sexualising or objectifying the video subjects. Sexualisation variables were coded the highest in the A4 Waist challenge, compared to the Spider leg and the Coin challenge. Overall, tight clothing was found in 41.11% of all videos collected across the three challenges, which became the most represented sexualisation variable in this study. This also draws similarities with thinspiration and fitspiration content with findings from Alberga, Withnell [35], which found that thinspiration and of fitspiration content tended to sexualise the subjects of images (in their study, 16.4% showed suggestive pose, 50.6% showed suggestive or partially clad clothing).

Objectification can influence the degree of sexualisation, which ultimately affects body image concerns. We theorised that body focused Douyin challenges would have greater objectification elements, and objectification of video subjects was approximated by the incidences of videos which did not show the full body of the subject. While the Spider leg challenge videos tended to be less objectifying in the sense that more of the body was shown (100% of videos showed the full body), most of them (60%) deliberately obscured the subject’s face or eyes. As the Coin challenge videos emphasised the collarbones, all of them did not show below the subject’s torso. Although the Coin challenge videos were coded as the most objectifying (47% not showing the face or eyes, and none showing the full body, legs or pelvis), this may be due to the nature of the challenge’s focus on collarbones. This is similar to findings from thinspiration studies, which found that Western thinspiration content tended to be objectifying by only focusing on specific parts of the body [35]. In agreement with this, Tiggemann and Zaccardo [39] found that more than half of the thinspiration content was objectifying in some degree. Overall, the findings of the current study in terms of sexualisation and objectification were similar to previous literature [35, 40, 61], and may suggest a similar mechanism in influencing body image concerns.

#### Absence of eating restriction and mental illness

The content analysis results indicated that the variables ‘Mentioning Mental Illness’ and ‘Dieting Restraint Messages’ were not found in the challenge videos, indicating that video subjects did not verbalise sentiments relating to mental illness or eating restriction in the videos. This was surprising given that previous literature has consistently reported positive correlations between internet use, body image concerns, restriction of eating and eating pathology [62, 63]. It is also well known that restriction of eating is one of the most pronounced risk factors for disordered eating and eating disorders [48]. It is possible that dieting was not mentioned due to Chinese collectivistic cultural values in the context of body image concerns [64–67]. Jung and Lee [66] suggested that upholding accepted appearance norms may be just an element of collectivistic cultures, to demonstrate adherence to social norms. An individual with such collectivistic cultural values may engage in harmful behaviours, such as restriction of eating, in order to fit in with their peers regardless of their body type. Although thinness may still be the ultimate goal, the importance of having the same or similar body type as the peers could be more dangerous. This suggests that investing efforts to achieve the ‘ideal’ body could be recognised as a collective struggle for a population, which could escalate into the normalisation of body image concerns, disordered eating behaviours, and other potentially harmful behaviours that include dieting and restrictions. However, it is noted that themes of ‘eating restraint’ was found from thematic analysis of the audio transcripts, captions, and text (see below).

Besides eating restriction, the mention of mental illness was also absent in all of the videos collected. The lack of mention for mental illness may be the result of cultural differences. Mental health is highly stigmatised in Chinese culture, and it is closely correlated with moral values and societal expectations [68, 69]. A person with mental health issues is more likely to face stigma and discrimination in China compared to other Western countries, and often result in more negative life outcomes, such as less social/familial support [69]. Therefore, not mentioning mental illness may be protective for both the video subjects and commenters to avoid unnecessary negative feedback.

#### Mention of celebrities

Mention of celebrities was coded in 6.67% of the A4 Waist, 6.67% of the Coin, and 3.33% of the Spider leg challenge videos. Even though mention of celebrity was not coded in many of the challenge videos, it was still important to include this variable because of Douyin’s unique operation system linking video subjects’ profit or other positive outcomes, such as fame, exposure, sponsorships, etc., with the popularity of their videos. By mentioning trendy celebrities, the video subjects are not only bringing the attention of celebrities’ fans to the videos but also garner views through relevant trends and potentially increasing their own fans [70]. For example, a famous singer in China, Hua Chenyu, was often mentioned in the Coin challenge videos because he participated in the challenge on multiple TV channels and TV shows. His participation video clips from these TV shows were then uploaded to Douyin, where the challenge became viral among his fans. From there, this challenge was able to reach a broader population not limited to Hua’s fans and became a national challenge. The participation of big-name singers and actors in these Douyin challenges was different from the participation of influencers or YouTubers in other studies [34]. The big-name singers and actors tend to have more fans and better reputations compared to individual influences that may only be known for certain populations. Therefore, mentioning or including big-name celebrities would help increase more engagement of the viewers, which promotes the videos and video subjects naturally. In addition, YouTube videos tend to be longer and allow the subjects to use disclaimers when engaging in potentially dangerous behaviours to protect the viewers and minimise the harm. Douyin is a short video platform, therefore, video subjects tend to jump right into the topic without disclaimers, which could potentially be more dangerous.

#### Challenging others

Challenging others was the other new variable for this study and was coded in 26.67% of the Coin, 6.67% of the Spider leg, and none of the A4 Waist challenge videos. Although not all of the challenge videos collected had challenged others, it was included as it is also unique to the nature of a Douyin Challenge. Video subjects use challenging others as a strategy to encourage engagements from the viewers and interact with the viewers in the hope to gain more impact on the Douyin platform. The operation system of Douyin allows the video subjects and viewers to interact in a way where viewers are presented with content that they are interested in, and video subjects are gaining profit or popularity through the engagement (likes, comments, completing challenges) of their viewers [70]. This operation system is potentially more dangerous than thinspiration/fitspiration, as the interactions are anonymous, and therefore, could reach more populations and faster due to its anonymous nature.

In addition, the challenges often encourage people to use the same music and the same filters to maintain the consistency of a challenge, which could play a role in normalisation of appearance-changing filter use. In fact, 96.6% of the Spider leg, 40% of the A4 Waist and 40% of the Coin challenge videos respectively were found to use the same music. Similar filters within the videos collected for each challenge were also found in 86.67% of the Coin, 70% of the A4 Waist, and 60% of the Spider leg challenge. When challenge videos use the same music and dramatic filters, it is difficult to differentiate whether the thinness of participants is attributed to their natural body or a filter that makes them look consistently thin and long-legged. Furthermore, the use of appearance-changing filters was found in the videos across the three challenges, as some of the videos seemed to be purposely blurry and colour corrected, which could be a way to cover blemishes and change appearance. With increased filter use associated with the challenges, users are increasingly exposed to a falsely thin and polished appearance, which is dangerous as these false images are becoming normalised and providing an unattainable benchmark for a normal body type.

Challenging others were coded in 26.67% of the Coin, 6.67% of the Spider leg, and 0% of the A4 Waist challenge videos. A video subject could challenge their viewers to engage in the activities verbally in the videos, by using captions, and by using hashtags. We theorised that whether and how a video subject decides to challenge their users depends on the nature of a challenge. If a challenge was more popular on social media platforms due to celebrities, TV shows, or other reasons, then video creators and subjects may be more likely to use challenge others as a strategy to stay on-trend and relevant. This theory could explain why challenging others was coded the highest in the Coin challenge videos, as many celebrities have participated in this challenge on TV shows. It is unclear why challenging others was coded in none of the A4 Waist challenge videos. A possible explanation may be that the A4 Waist challenge is an older challenge from 2016 and people have eventually moved on from actively participating in this challenge at the time of video extraction. Other factors that may affect the coding of challenging others need to be investigated. We theorised that potential research factors may include: (1) the age groups or populations that a challenge is targeted to, (2) the impact factor (ie., how influenceable a person/subject is) of the celebrities who participated in a challenge, (3) the impact factor of the media platforms, (4) where the challenge is showcased, and (5) cultural factors that impact the value of body/beauty traits promoted by a challenge.

### Thematic analysis

#### Audio transcript, captions and text

The audio transcripts as well as video captions and text provided a deeper insight into the behaviours of video creators, beyond the visual content of subjects, as some creators offered commentary while including content of others. Creators focused heavily on appearance ideals, illustrated in the subthemes Challenge as a Reflection of Ideal, Collective Struggle to Achieve Ideal, Methods to Achieve Ideal, Thin is Good, and common references to Muscular and Posture. The challenges provided a benchmark of ideal appearance, equating success with achievement of beauty standards. Fans want popular subjects to participate in the challenges to prove their beauty (Pressure to Participate), and many subjects express happiness in succeeding (I’m Happy I Succeeded), an indication that they have attained the benchmark. The most prominent appearance ideal mentioned is thinness, which is further glorified by being linked to romantic success and health. These ‘positive’ connotations are reinforced with the results of the content analysis, with mostly thin subjects objectifying their bodies and portraying suggestive connotations.

As noted above, a higher proportion of the A4 Waist challenge videos contained voiceover content by the video creators, not the speech spoken by the video subjects themselves. Accordingly, there was a higher proportion of exercise videos under the A4 Waist challenge. Several of these videos provided educational guides from supposedly certified doctors or personal trainers to help viewers lose weight and to succeed in the challenges. This reflects the findings of Ratwatte and Mattacola [34] highlighting a common ‘authoritative’ theme of content subjects, which is concerning as these subjects legitimise the information and messages that they spread. The subtheme Beauty Reflects Effort echoes findings of previous fitspiration and thinspiration studies that attractiveness (and therefore challenge success) is a motivating factor to exercise [35, 39, 42]. In accordance with our finding, Alberga, Withnell [35] noted that the encouragement to exercise for appearance-related reasons has been previously linked to negative body image [71, 72], disordered eating [73] and depressive symptoms [74].

To conclude, the content analysis described above demonstrated that most subjects were coded as thin, a small number were coded as average, but none had larger bodies or were coded as overweight. Combined with their themes of encouraging everyone to complete exercises together and lose weight (Collective Struggle to Achieve Ideal), they portray that challenge success is more easily achieved. They also limit the range of body types that are idealised, which is shared with thinspiration and fitspiration studies [35, 39], and can result in body dissatisfaction.

#### Video comments

Thematic analysis of comments provided an insight into the impact of challenge videos on viewers. The four major themes of the video comments were Praise of Challenge, Praise of Video Subject, Criticism of Challenge, Criticism of Video Subject. More comments were found to fit the theme Praise of Challenge than Criticism of Challenge. Those who criticised expressed concern about the impact of the challenge on culture. However, the most frequent comments across all three challenges were tagging and challenging others. In comparison, previous thematic analysis studies on thinspiration and fitspiration did not note any subjects or commenters actively challenging other users [34, 37]. Furthermore, Douyin/TikTok differs from other social media platforms in that users are passively shown videos that may be of relevant interest to them, rather than users actively searching hashtags or specific accounts as with thinspiration or fitspiration content. This wider exposure of such videos is a characteristic of these ‘challenge’ trends, and particularly concerning when considering the implications of the increased messaging and associated influence on body dissatisfaction.

Many commenters engaged in negative self-comparison. Commenters directly expressed a desire to diet after watching such videos, and many sought exercise advice, which is in line with many previous studies suggesting that repeated exposure by social media to unrealistic ideals of beauty in females is a risk factor for body dissatisfaction, weight concern, thin-ideal internalisation and disordered eating behaviours in women [2, 5]. This is concerning given that the content analysis results showed no mention of eating restriction indicated by the variable ‘Dieting Restraint Messages’; it suggests that the portrayal of a certain body type is enough to encourage the desire to restrict eating. Furthermore, analysis of the comments supports the findings of the experimental study of Jackson et al. [31], who found that body dissatisfaction increased when viewing images of thin women participating in the A4 Waist challenge as opposed to a regular photo. These are preliminary suggestions that exposure to challenges may possibly be more harmful than thinspiration or fitspiration exposure.

Within both the themes Praise and Criticism of Video Subject, there were more comments about Physical Traits of the subject compared to their Non-Physical Traits, suggesting the nature of the challenges place greater fixations on certain body parts, which reflects the findings of our content analysis of high levels of objectification in the videos. In accordance with this finding, an experimental study by Tiggemann and Barbato [75] looking at the relationship between photos and positive comments found that viewers who read appearance-related comments also reported greater body dissatisfaction than place-related comments despite being shown identical photos. Similarly, Kim [76] found that reading body-favourable comments lead to greater idealisation of body imagery, while body-unfavourable comments lead to lower idealisation. Thus, despite being positive, Praise of Physical Traits may contribute to a greater sense of dissatisfaction, and the quantity of body-praising comments outweighs the critical ones which may reduce idealisation.

##### Criticism of video subject

While no studies directly investigate the effects of negative social media comments on video subjects, the Tripartite Influence Model [53] provides a conceptual framework for development of body dissatisfaction and disordered eating originating from three core sources of influence—family, peers and the media. Other studies highlight that peer feedback influences can affect weight-loss desire [77], internalisation of ideals and body image disturbances [78]. However, since the publication of many of these works, social media has risen to become a dominating sociocultural influence. Roberts et al. [79] adapted the model to include social media as a fourth source of influence and found that appearance pressures from social media lead to appearance comparison and thin-ideal and muscular-ideal internalisation. Thus, through peer feedback and social media, which both fit into the Tripartite Influence Model, critical comments about social media content subjects can act as pressures on body dissatisfaction. Considering the effects in the other direction, ie. content on viewers, reflected in the subtheme Negative Self-Comparison, Fardouly and Vartanian [4] suggest that the ability to view media posted by peers and engage in peer content can effectively cause greater internalisation of thin-ideals and appearance comparisons. In addition, another study found that Asian women were found to have the highest levels of levels of appearance pressures and internalisation compared to Black women and Latina women, which may suggest more detrimental effects from social media in Asian populations [56]. This highlights the potential bi-directional negative influence on both creators and viewers of the challenge videos, especially in Asian populations.

### Implications and future directions

The current study on Douyin challenges has several theoretical and clinical implications that are important to outline in hope to inform public health and guide future research studies.

First, this study has added unique cultural values to the theoretical association of social media use and body image concerns. Research has consistently reported body dissatisfaction being a strong risk factor for eating pathology, eating restriction, and the onset of eating disorders [47, 48]. Therefore, Douyin challenges videos that promote body dissatisfaction through thin body presentations and unrealistic body expectations may be dangerous as they could play a role in the development of eating disorders. Although dieting has been reported as a strong predictor for eating pathology in previous literature, interestingly, dieting informed by the variable ‘Dieting Restraint Messages’ was not coded in any of the challenge videos collected in current study [47, 48]. We theorised that the lack of mention for eating restriction, or any forms of dieting may suggest the normalisation of dieting and that body image concerns are identified as collective struggles. Such normalisation of unhealthy body standards may be due to the collectivistic cultural values in China, which implies that the same trends on Douyin or other short-video platforms could be more dangerous for Asian populations compared to other ethnic groups.

The social expectations and peer pressure to look a certain way as everyone else may also be a result of such unique cultural values. In addition, it has also been reported that tags or language used in pro-eating disorder communities have constantly been deleted and removed to protect vulnerable individuals, which facilitates the evolution of language used to refer to certain pro-eating disorder content over time [80]. The language used to refer to a specific challenge trend could change overtime, which may help explain why the coding of older challenges (eg., Coin challenge and the A4 Waist challenge) were different from the newer challenges (eg., the Spider leg challenge). Hence, more research is needed to investigate what and how cultural differences play a role in the associations between social media use and body image concerns using a longitudinal perspective, as well as monitor their evolution over time.

Clinically, developing body dissatisfaction because of cultural values suggests that Asian populations are as vulnerable to unhealthy body-focused trends and as susceptible to developing eating disorders compared to other populations. As implicated, attention should be focused on not only Chinese populations, but also other Asian countries or cultures that may also share similar collectivistic cultural values as Chinese culture. For example, Mukbangs, eating shows that originated in South Korea where video subjects consume large amounts of food on camera, have been reported to be associated with restrictive eating and loss-of-control eating, and overall mental health problems [81, 82]. For this reason, clinicians need to be aware of whether their patients, especially the ones with Asian cultural backgrounds, engage in social media trends associated with body image concerns and eating disorders.

Considering the above, to prevent the worsening of body image concerns, disordered eating behaviours, and new onset of eating disorders, it may be helpful to monitor trends on social media and short-video platforms that are body-focused. Our current study also addressed special considerations when assessing the associations of social media use and body image concerns in Asian population. It would be important to teach the users to recognize cultural differences, as some cultural values may also interact with the association of social media trends and body image concerns. In addition, there is a need for social media literacy training, which includes teaching users that the body of video subjects in these challenge videos may be purposefully altered to meet unrealistic body expectations.

### Limitations and strengths

The study was affected by several limitations, which are important to outline. The primary limitations of this study include the cultural context of the coders and the limited sample size of videos and comments extracted in the content analysis. First, the cultural context of each of the coders for both the content and thematic analyses informed their coding decisions, which may misrepresent the sentiments expressed by video subjects and commenters. While the three coders (SH, JG, and VS) are of Chinese heritage and have competency in the Chinese language, they are not currently located in China and received some parts of their education in Australia and the United States. Every effort was made to interpret meaning through a Chinese cultural lens; however, it must still be noted that the thematic and content analyses were conducted by coders with a Western-biased understanding of social media use, landscape, and lifestyle integration.

Second, the relatively small sample size of videos (N = 90) and comments extracted (N = 5508) is another limitation in that the coded videos and comments may not be an accurate representation of the population of videos and may not reflect the trend at large. It is critical to note that many of the variables outlined in Table 3 had less than five counts, which hindered statistical analyses. Therefore, our content analysis statistics were kept descriptive, and future studies ought to assess more videos to allow for the possibility to compare across the challenge using statistical tests. The popularity peak of all three challenges occurred at different times, even different years; for example, the A4 Waist challenge originated in 2016 [31]. Accordingly, the age of some of the challenges analysed in the current study (in particular, the Coin challenge which originated at the latest in 2015) meant that videos which were once popular a few years ago at the height of the challenge’s popularity may have since been deleted. Furthermore, the temporal difference between the video’s creation and the present study may be enough to shift the sociocultural context within which the challenges were viewed.

Lastly, a final limitation was potential errors in extracting the comments. Python was used to extract the comments into an Excel file format, the visual impact of some emojis were limited.^6^ The challenge in both cases was to accurately extract meaning from a visual symbol or a description of one.

Despite these limitations, the study also had several notable strengths. Its primary strength was the application of an inductive, mixed methods approach to the analysis of a novel set of data (Chinese social media, or Douyin videos specifically). Specifically, where literature is scarce, the use of inductive research methods allowed the data to guide research hypotheses without preconceived biases. In addition, the study was able to consider both Chinese and Western cultural contexts comparatively due to the coders’ language competencies in both English and Chinese. Furthermore, analysis of comments demonstrated it can be a rich source for investigating the impacts of social media content on viewers.

## Conclusion

This study aimed to explore the relationships between Douyin body challenge videos and body dissatisfaction in both video creators and viewers. Overall, the trending challenges contain content of concern with regards to body image and risks for disordered eating. The nature of the challenges, elements of objectification and sexualisation, thin-ideal messaging and unnatural filtered appearances contribute to negative and problematic attitudes towards body image. Thematic analysis of users’ comments revealed that viewing challenges can lead to greater body dissatisfaction, in addition, viewers can participate and objectively compare themselves. Moreover, the widespread nature of challenge through users’ participation and tagging, or digitally by the Douyin platform itself, means the risk of body dissatisfaction or disordered eating may be greater than other social media trends with similar risks. The study hopes to inform further research on short-video platforms similar to Douyin, including TikTok or YouTube Shorts or Instagram Reels, or Chinese social media, or challenge videos specifically.

## Data Availability

All data analysed during this study are included in this article (tables/figures).

## Footnotes

1 The codes refer to individual videos. SL denotes a Spider Leg video, and A and C denote A4 Waist and Coin challenge videos respectively.

2 A well-known Chinese singer.

3 A well-known Chinese actor.

4 A well-known Chinese actress.

5 666 = code of “Cool!” emoji in the Chinese keyboard.

6 Some emojis were extracted in their original form, while others were extracted as alternative text. For example, a smiling face emoji may appear in its extracted form as an emoji, others appeared as ‘[smile]’.
